# Hip arthroscopy enables classification and treatment of precollapse subchondral insufficiency fracture of the femoral head associated intra-articular pathology

**DOI:** 10.1007/s00167-017-4722-4

**Published:** 2017-09-23

**Authors:** Soshi Uchida, Moriyuki Noguchi, Hajime Utsunomiya, Shiho Kanezaki, Toshiharu Mori, Dean K. Matsuda, Akinori Sakai

**Affiliations:** 1 Department of Orthopaedic Surgery, Wakamatsu Hospital of the University of Occupational and Environmental Health, Kitakyushu, Japan; 2 Department of Orthopaedic Surgery, Faculty of Medicine, University of Occupational and Environmental Health, Kitakyushu, Japan; 3 DISC Sports and Spine, Marina Del Rey, CA USA

**Keywords:** Subchondral insufficiency fracture of the femoral head, Hip arthroscopy, Acetabular labral tear, Femoroacetabular impingement, Arthroscopic internal fixation, Femoral head fracture

## Abstract

**Purpose:**

The purposes of this study were to investigate (1) the clinical, radiographic and arthroscopic presentation of patients with subchondral insufficiency fracture of the femoral head (SIFFH) and (2) the outcomes following arthroscopic treatment with internal fixation using hydroxyapatite poly-lactate acid (HA/PLLA) threaded pins and concomitant arthroscopic treatment of associated findings.

**Methods:**

Nine patients (median age 49.0 years, range 43–65, five female and four male patients) with SIFFH who underwent arthroscopic treatment with labral repair, capsular closure and internal fixation of SIFFH using HA/PLLA pins were retrospectively reviewed. Inclusion criteria were adult patients with precollapse SIFFH with minimum 1-year follow-up (median follow-up 30.0 months, range 12–56).

**Results:**

Acetabular labral tears were observed in all patients. The median BMI was 24.3 kg/m^2^ (range 20.1–31.8). Clinical presentations and radiographic measurements demonstrated mixed type FAI in six patients, borderline developmental dysplasia in two patients and pincer type FAI in one patient. The median MHHS significantly improved from preoperatively (67.1, range 36.3–78.0) to post-operatively (96.8, range 82.5–100; *p* = 0.001). The median NAHS significantly improved from preoperatively (34.0, range 17–63) to post-operatively (78.0 range 61–80; *p* = 0.001).

**Conclusion:**

SIFFH is associated with bony deformities and labral tears. Precollapse SIFFH can be treated with bioabsorbable pin stabilization of unstable lesions and treatment of associated intra-capsular pathology in those with stable lesions as determined by a new arthroscopic classification system with promising early outcomes.

**Level of Evidence:**

IV.

## Introduction

Subchondral insufficiency fracture of the femoral head (SIFFH) is recognized as an uncommon cause of acute hip pain and is differentiated from osteonecrosis of the femoral head. A recent study reports SIFFH occurring in military recruits and patients with osteoporosis [21]. Patients usually suffer from the acute onset of pain following minor injury and prolonged untreated SIFFH can result in rapidly progressive osteoarthritis of the hip joint [5, 21]. However, the pathomechanism of SIFFH still remains unknown.

In its precollapse stage, the diagnosis of SIFFH can be established with magnetic resonance imaging (MRI).

Magnetic Resonance Imaging (MRI) may distinguish SIFFH from osteonecrosis, osteochondritis dissecans (OCD) and transient osteoporosis of the hip (TOH). On MRI, SIFFH displays diffuse bone marrow oedema pattern on STIR and a low-intensity band on T1-weighted images. The shape of the low-intensity band in SIFFH is typically discontinuous, irregular, serpentine and parallel to the articular surface. This differentiates SIFFH from other hip pathologies, which may have different prognoses (e.g. osteonecrosis often progresses to femoral head collapse and degenerative osteoarthritis with relatively poor prognoses, whereas TOH often resolves with conservative treatment) [10]. In contrast, Pape et al. [15] have shown in a review article that SIF may be the initiating factor of what was formerly believed to be spontaneous osteonecrosis of the knee (SPONK). It is possible that SIF and osteonecrosis can be stages of the same disease even in hip joint.

SIFFH often progresses to subchondral collapse of femoral head predisposing to secondary osteoarthritis [22]. Several reports demonstrate the surgical treatment of SIFFH. Yamamoto et al. [21] described trochanteric rotational osteotomy as effective in the treatment of younger patients with SIFFH since their fractures are typically in the anterosuperior region of the femoral head. Several studies also have reported that the majority of older patients with subchondral collapse have been treated with total hip arthroplasty (THA) for secondary osteoarthritis [9, 21].

The early phase of SIFFH was typically managed with conservative treatment including non-weight bearing with crutches to minimize adverse mechanical stress against injured femoral head and non-steroidal anti-inflammatory agents [21]. Although some patients improve with conservative treatment, many cases progress to femoral head collapse despite even prolonged non-weight bearing. A minimally invasive treatment option for precollapse recalcitrant SIFFH has not been reported. As little is known of SIFFH, hip arthroscopy may aid its assessment as well as detection of associated intra-capsular pathology.

Moreover, hip arthroscopy enables minimally invasive internal fixation of osteochondral lesions and fractures of the femoral head. Arthroscopic fragment fixation using hydroxyapatite poly-lactate acid (HA/PLLA) composite offers a less invasive and effective treatment for intra-articular osteochondral lesions in a variety of joints. Uchida et al. [19] demonstrated that excellent healing and clinical outcomes for the patients with osteochondritis dissecans of the elbow were demonstrated following arthroscopic fragment fixation with HA/PLLA threaded pins. We hypothesize that hip arthroscopy is a useful tool for assessing and treating patients with precollapse SIFFH.

The purposes of this study were to investigate (1) the clinical, radiographic and arthroscopic presentation of patients with SIFFH and (2) the outcomes following arthroscopic treatment with internal fixation using hydroxyapatite poly-lactate acid threaded pins and concomitant arthroscopic treatment of associated findings.

## Materials and methods

**Fig. 1:**
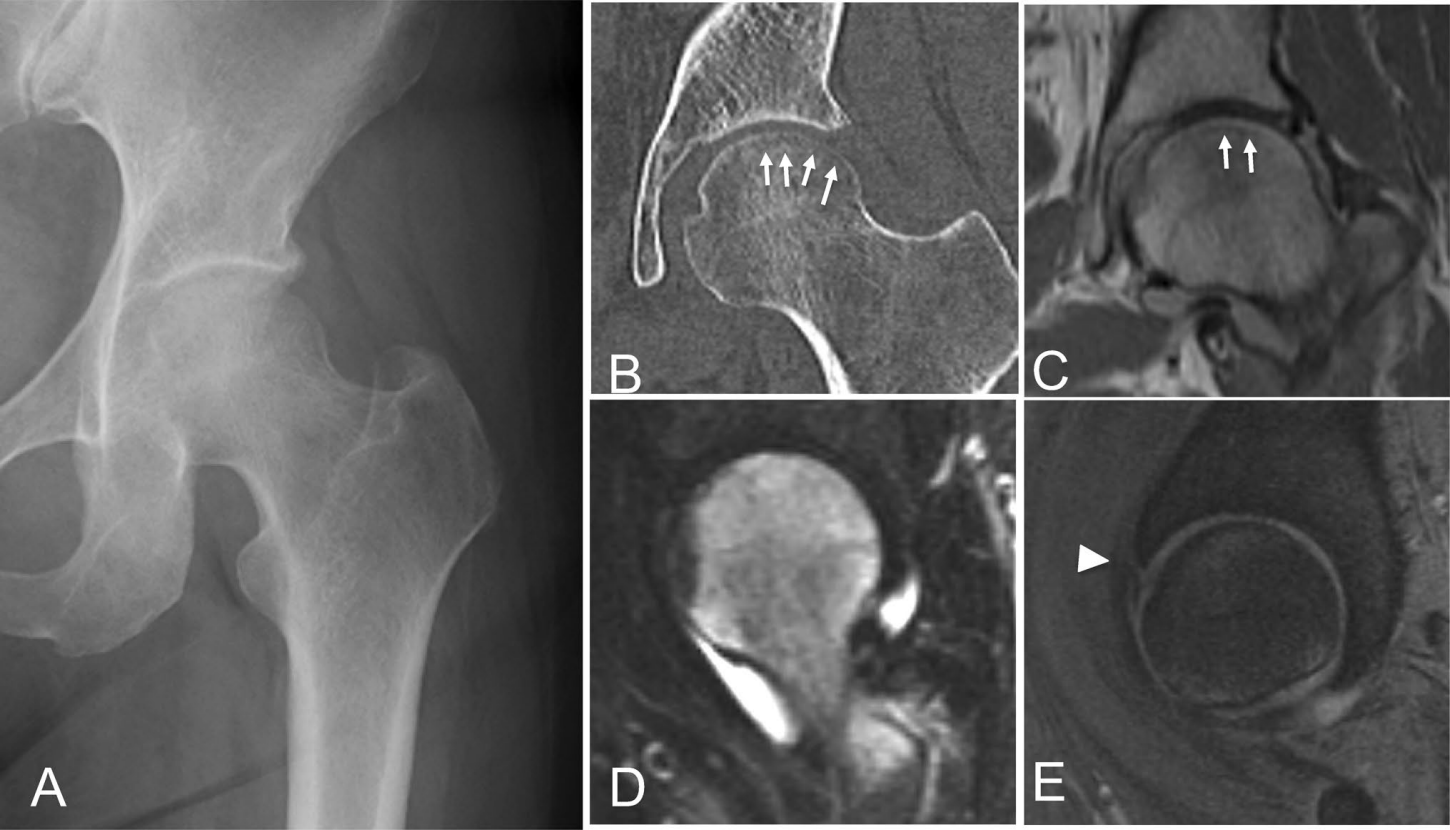
A 45-year-old female presented with 4-month history of increasing left deep groin pain. She had a limited range of motion of 90° of flexion and 15° of internal rotation. She also had a positive anterior and posterior impingement sign. **a** An anterior–posterior (AP) pelvic view shows LCE angle of 30° and intact Shenton’s line. There was no visible fracture line or loss of sphericity of the femoral head. **b** Computed tomograph coronal view shows curved hair line paralleled to articular surface of the femoral head. **c** Proton density coronal views show lo-intensity band parallel to subchondral area along femoral head. **d** Oblique sagittal MRI short-time inversion recovery (STIR) view shows diffuse high-intensity area suggesting bone marrow oedema pattern in entire lesion of the femoral head. **e** T2-star-weighted oblique axial view shows anterosuperior labral tear (arrow)

The clinical records of 623 patients who underwent hip arthroscopic surgeries between 2009 and 2016 were retrospectively reviewed. Inclusion criteria were adult patients with precollapse SIFFH concurrent with borderline developmental dysplasia of the hip (BDDH), FAI and labral tears. We performed arthroscopic antegrade internal fixation with HA/PLLA pins, labral repair and femoral osteoplasties. No patients required open procedures. The diagnosis SIFFH was based on a comprehensive history of hip pain that began without any history of major trauma, normal radiographs with regard to the femoral head (Fig. 1a), a fracture line parallel to the articular surface on computed tomogram (CT) (Fig. 1b), diffuse femoral head bone marrow oedema pattern on MRI and subchondral low-signal-intensity band on Proton or T1-weighted MRI that was irregular, serpiginous and parallel to the articular surface (Fig. 1c). Oblique sagittal or coronal views also showed diffuse femoral head bone marrow oedema pattern (Fig. 1d) and acetabular labral tear (Fig. 1e) [21]. Patients with osteonecrosis and osteochondritis dissecans of the femoral head were excluded.

One patient was diagnosed as SIFFH at 12 months due to persistent pain after internal fixation of a femoral neck fracture and was excluded because of prior ipsilateral hip surgery. Nine patients met the inclusion criteria at minimum 12-month follow-up (median, 30.0 months, range 12–56).

Regarding other bony abnormalities associated with SIFFH, including FAI and BDDH, clinical inclusion criteria were groin pain for more than 3 months, restricted hip ROM (flexion < 105° and/or restricted internal rotation in flexion < 20°), positive flexion abduction external rotation test (FABER) and a positive anterior impingement test. The hip dial test was utilized to detect anterior capsular laxity. It is performed with the patient supine and the hip in neutral extension. The leg is internally rotated and then released to allow for relaxed external rotation. A positive test is defined as greater external rotation of the affected hip than that of the contralateral hip [4].

Radiographic evidence of a cam deformity (alpha angle > 55° or head–neck offset < 8 mm on at least one radiographic view or computed tomography CT or magnetic resonance imaging MRI), [2], and/or pincer deformity, defined as lateral CE angle (LCEA) from well-centred AP pelvic radiograph > 25° plus crossover sign, coxa profunda, or posterior wall sign, and/or prominence of the ischial spine sign. Patients with LCEA ranging from 20° to 24° were diagnosed as BDDH. Physical examination was also performed to evaluate impingement and instability of the hip joint. Exclusion criteria included patients who had only non-surgical treatment, femoral head osteonecrosis or follow-up period less than 1 year.

### Radiographic assessment

The radiographs of all nine patients were assessed using a picture archiving and communication system (PACS) on anteroposterior view of the pelvis, cross-table lateral and modified Dunn view (45° of hip flexion and 20° abduction in neutral rotation) [3]. The lateral centre edge angle (LCEA), Tönnis angle and Sharp’s angle were measured in AP view with the patient in supine position, and femoral neck shaft angle was measured in AP view with the patient in standing position. Shenton’s line was measured on a standing pelvic AP view, and the alpha angle on cross-table lateral view or modified Dunn view [3]. LCEA was formed by the intersection of a line drawn through the centre of the femoral head and extending to the lateral edge of the sourcil (the dense bone along the lateral edge of the weight-bearing region of the acetabulum), and a line perpendicular to one bisecting the two femoral head centres [20].

The radiographic measurements were utilized as follows: LCEA to define the lateral coverage of acetabulum, Tönnis angle as a measure of acetabular inclination, Sharp’s angle as a measure of acetabular index and alpha angle as a measure of cam deformity. Vertical centre anterior (VCA) angle on false profile view in standing position was used as a measurement of anterior coverage [1, 12].

#### Operative technique

**Fig. 2:**
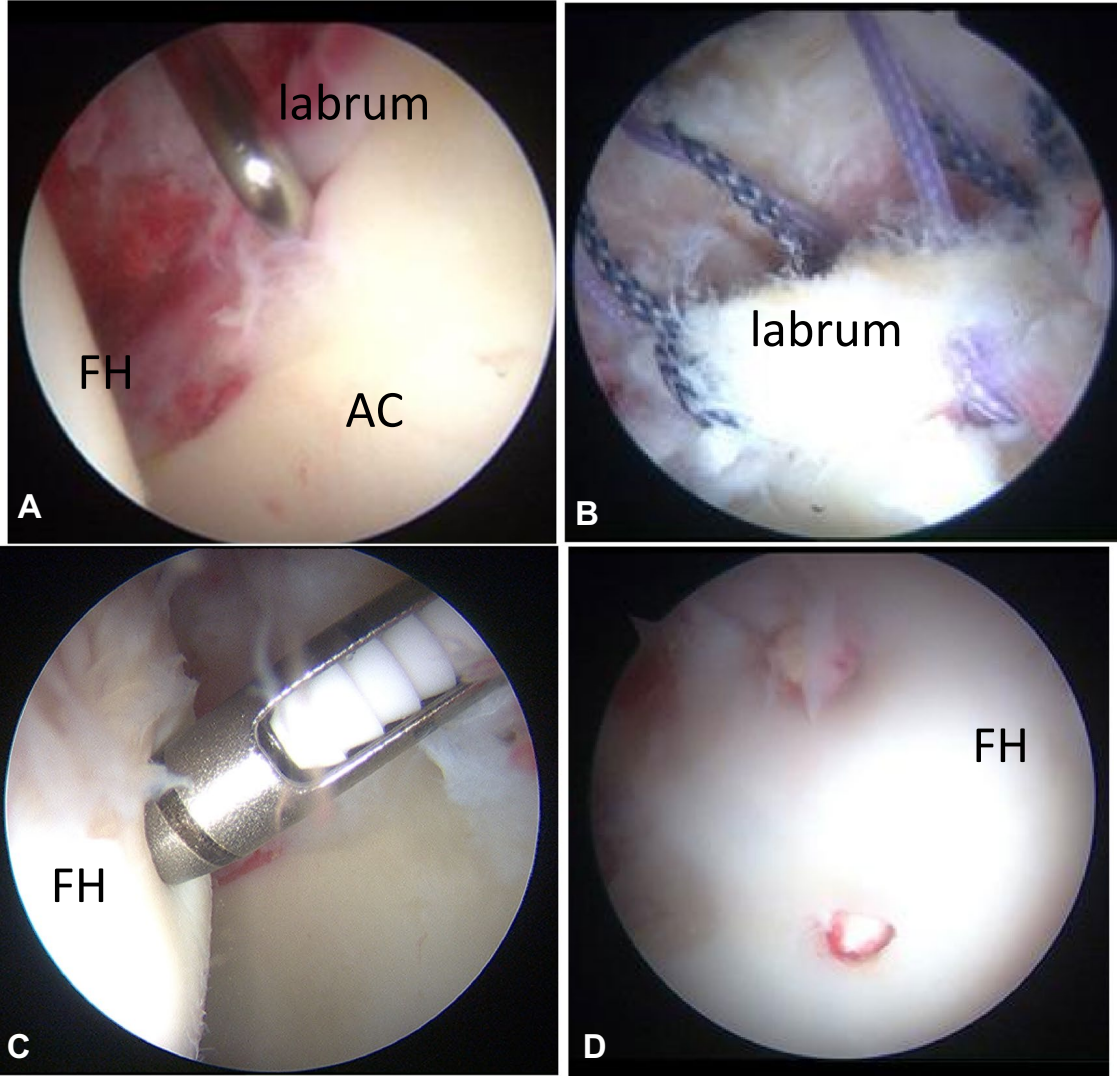
Arthroscopic findings **a** supine arthroscopic view from ALP shows anterosuperior labral tearing. **b** Supine arthroscopic view from ALP shows labral repair via four sutures with two suture anchors, **c** Supine arthroscopic view from ALP shows internal fixation of SIFFH with Superfixorb thread pins introduced through drill guide through PMAP. **d** Viewing from PMAP shows stable SIFFH after fixation with HA/PLLA thread pins. Fixation of SIFFH lesion with four PLLA-HA pins (dia2 × 15 mm)

Supine hip arthroscopy under general anaesthesia was performed with well-padded peroneal post on a traction table. An anterolateral portal (ALP) and midanterior portal (MAP) were established. After inter-portal capsular release, intra-articular pathologies including SIFFH lesion, labral tearing and any associated pathologies were assessed. If acetabular labral tear and/or associated cartilage delamination was observed (Fig. 2a), a radiofrequency probe was utilized to smooth frayed or small flaps of labral tears and/or labral refixation was performed with bioabsorbable suture anchors (Fig. 2b).

During central compartment procedures, the SIFFH lesion was arthroscopically assessed. The location of SIFFH lesion was assessed using the geographical zone method [7], and a new classification system was established for SIFFH (Uchida classification): Grade 0—normal; Grade I—precollapse; Grade IA—stable to probing, normal cartilage; Grade IB—stable to probing, cartilage fissure or fibrillation; Grade IC—unstable to probing, partial discontinuity of cartilage; Grade II—subchondral collapse; Grade IIA—no arthrosis. Unstable to probing, partial discontinuity and collapse of cartilage; Grade IIB—mild arthrosis with Cartilage degeneration—collapse of cartilage and subchondral bone; Grade III—major arthrosis (Tonnis 3) with complete chondral and subchondral collapse (Table 1) (Fig. 2c).

**Table 1:** Uchida Classification system for SIFFH

	Subchondral bone	
Grade 0	Normal	
Grade I	Precollapse	
Grade IA		Stable to probing cartilage normal
Grade IB		Stable to probing cartilage fissure or fibrillation
Grade IC		Unstable to probing, partially discontinuity of cartilage
Grade II	Subchondral collapse	
Grade IIA		No arthrosis unstable to probing, partially discontinuity of cartilage
Grade IIB		Mild arthrosis with cartilage degeneration
Grade III		Complete chondral collapse and major arthrosis (Tonnis grade III)

After arthroscopic assessment, the fixation site was prepared before actual arthroscopic procedures. The proximal midanterior portal (PMAP) was established to access the SIFFH lesions. If the SIFFH lesion was located in the weight-bearing area, the hip was hyperextended 10°. If the SIFFH was determined to be grade IC or 2A (Fig. 2c), arthroscopic fixation of the SIFFH lesion was then performed using HA/PLLA threaded pins (Super Fixorb 30 thread pin; Takiron Co., Ltd Osaka, Japan). A tamp (DePuy) was utilized to insert the HA/PLLA threaded pins. Preoperatively, the length of the Super Fixorb pin had been determined by measuring the depth of the SIFFH lesion using coronal and/or sagittal multiplanar reformation (MPR) computed tomography (CT) images. A length twice the distance from SIFFH fracture line to articular surface was selected.

A 2-mm drill guide was introduced through the PMAP under arthroscopic guidance (Fig. 2c). The guide hole was initiated with a custom-made dilator bit. Drilling was advanced to the desired depth using a 2.0-mm Kirschner wire. The dilator was inserted into the drill guide and was tapped to the desired depth. Next, the HA/PLLA pins were advanced through the drill guide with a delivery tamp to fixate the SIFFH lesion (Fig. 2c, d). Two or three HA/PLLA pins were typically required, especially for unstable (Grade IC) SIFFH lesions. Arthroscopic probing was then done to confirm secure osteochondral fixation (Fig. 2d).

After releasing traction, cam osteochondroplasty was performed using a motorized round burr when indicated. Arthroscopic capsular repair was then performed as previously described [17].

### Post-operative rehabilitation protocol

**Fig. 3:**
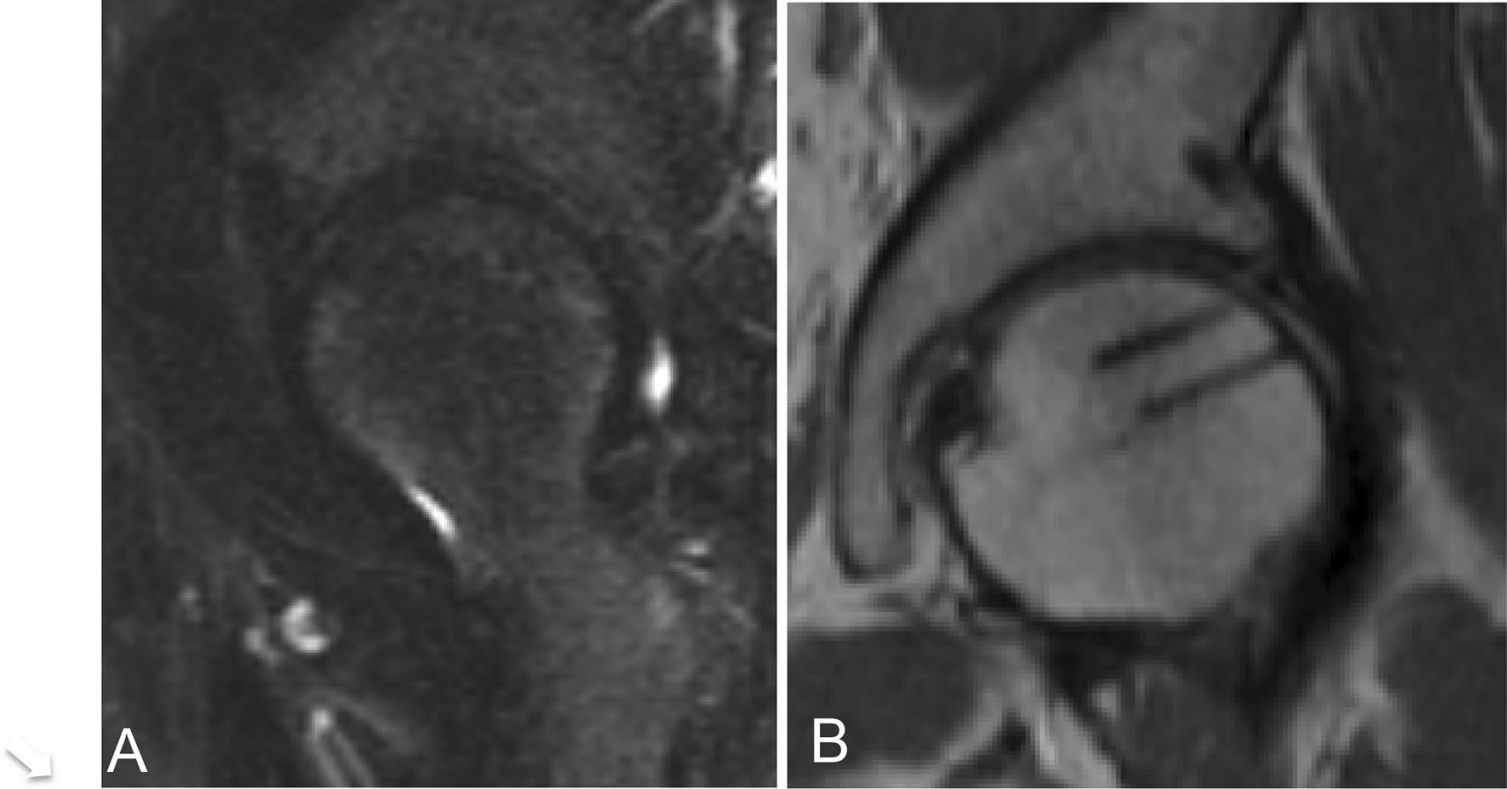
MRI findings **a** oblique sagittal T2-weighted fat saturation MR views showed complete disappearance of bone oedema pattern at 1 year after surgery. **b** Coronal proton density weighted MR view shows disappearance of fracture line and low-intensity bars indicating HA/PLLA threaded pins (arrows)

Gentle passive range of motion (ROM) exercise was initiated during the first week. The patient remained non-weight bearing for 4 weeks. MRI at 4 weeks after surgery showed diminished bone marrow oedema. After 4 weeks, weight bearing was gradually increased with full weight bearing by 8 weeks after surgery. Endurance strengthening began at 16 post-operative weeks after range of motion and lower extremity control was achieved. Throughout this phase, impact aerobic conditioning was prohibited. At 1 year after surgery, all patients had returned to work or activity. MRI revealed resolution of bone marrow oedema pattern, healed labrum, and no evidence of fracture line (Fig. 3a, b).

### Clinical outcome

Data, including but not limited to patient-reported outcome (PRO) scores, were collected during office visits with the operating surgeon. PRO scores included the modified Harris hip score (MHHS) and the non-arthritis hip score (NAHS) and were obtained preoperatively and at final follow-up.

This study was approved by local institutional review board (University of Occupational and Enviornmental Health, approval number: H27-050).

### Statistical analysis

Wilcoxon rank sum test was used to assess the difference in scores obtained preoperatively and post-operatively by use of SPSS Japan software, version 15. Statistical analysis was completed with data reported as median (range) and *p* < 0.05 defined as significant.

Power analysis using the data of initial five cases demonstrated minimum sample size of four patients to show the significant difference between preoperative and post-operative modified Harris Hip Score (Wilcoxon ranked sum test, *α* = 0.05, effect size = 2.7, actual power = 0.91).

## Results

Nine patients met the inclusion criteria at minimum of 12-month post-operative follow-up. No patients had prior history of hip surgery.

The median age was 49 years old (range 43–65) at the time of surgery. There were five female patients and four male patients. There were five right hips and four left hips. The median BMI was 24.3 kg/m^2^ (range, 20.1–31.8). The median period from symptom onset to surgery was 6.0 months (range 3–40 months). Relevant preoperative hip examination findings are summarized in Table 2. Preoperative radiographic findings are summarized in Table 3. Of note, mixed FAI was present in six patients, borderline developmental dysplasia (BDDH) in two patients and pincer FAI in one patient. All radiographic data are shown in Table 3.

**Table 2:** Patient demography and clinical presentation

Age	Median 49 (43–65)
Gender	4 male and 5 female
Side	5 right hips and 4 left hips
BMI	Median 24.3 (20.1–31.8)
Time to surgery	Median
Follow-up period	Median 29.5 (12–56)
Physical examination	
AIT	9 of 9 positive
PIT	7 of 9 positive
Dial test	9 of 9 positive

*N* number, *AIT* anterior impingement test, *PIT* posterior impingement test

**Table 3:** Summary of radiographic data

Bony abnormalities	Mixed type FAI: 6, pincer type FAI:1, borderline DDH, 2
LCE angle	32° (range 23°–47°)
Sharp angle	40° (range 30°–44°)
Tonnis angle	5° (range −8° to 14°)
VCA angle	31° (range 25°–44°)
Alpha angle	70° (range 43°–87°)
Tonnis classification grade of OA	4 hips in grade 0 and 5 hips in grade 1

*LCE* lateral centre edge, *VCA* vertical centre anterior, *OA* osteoarthritis

Intra-articular findings including SIFFH lesions and status of acetabular articular cartilage, labrum and ligamentum teres are shown in Table 4. Acetabular labral tears were present in all patients. Acetabular cartilage delamination was found in all patients (MAHORN grade I in two cases, grade II in one case and grade III in three cases). Importantly, the cartilage lesions were classified by the new SIFFH classification scheme based on arthroscopic observation (Grade 1A in two cases, Grade IB in one case, Grade 1C in five cases, Grade 2A in one case). The median MHHS significantly improved from preoperatively (67.1, range 36.3–78.0) to post-operatively (96.8 range 82.5–100; *p* = 0.001). The median NAHS significantly improved from preoperatively (34.0, range 17–63) to post-operatively (78.0 range 61–80; *p* = 0.001). No radiographic osteoarthritic progression was observed by Tonnis grade. Post-operative MRI at 1 year after surgery showed bone marrow oedema completely resolved and SIFFH lesion successfully healed in all patients (Fig. 3b, c). There were no complications, revision surgeries or conversion arthroplasties following initial hip arthroscopic surgeries.

**Table 4:** Arthroscopic findings and procedures in each SIFFH group (acetabular labral pathology, ligamentum teres)

SIFFH group	Number of cases	Zone	Procedure	Number of HA/PLLA pinsa	Delamination	Labral tear	Labral treatment	Ligaments teres pathology
I A	2	3L: 2 cases	Left in situ: 2 cases	–	MAHORN 0: 1 case	Partial: 1 case	Repair: 1 case	Synovitis: 2 cases
					MAHORN 1: 1 case	Frayed: 1 case	Reconstruction: 1 case	
I B	1	2S-2M-3S-3M: 1 case	Left in situ: 1 case	–	MAHORN 2: 1 case	Partial: 1 case	Repair: 1 case	Frayed: 1 case
I C	6	2S-3S: 4 cases	HA/PLLA: 6 cases	2 pins: 3 cases	MAHORN 1: 1 case	Partial: 1 case	Repair: 5 cases	Intact: 1 case
		3S: 1 case		3 pins: 2 cases	MAHORN 2: 1 case	Frayed: 2 case	Debridement: 1 case	Synovitis: 2 cases
		2S-2L: 1 case		4 pins: 1 cases	MAHORN 3: 4 case	Complete: 3 cases		Partial: 2 cases
								Complete: 1 case

ITT; iliotibial tract, HA/PLLA; hydroxyapatite poly-lactate acid

## Discussion

The main findings of this study are that the arthroscopic internal fixation of precollapse SIFFH with HA/PLLA thread pins in concert with arthroscopic treatment of associated bony deformities and labral tears provided clinical benefit. Union of SIFFH with resolution of bone oedema occurred in all patients. Moreover, none progressed to discernable femoral head collapse or worsening of radiographic osteoarthritis for the duration of the study. Hence, our hypothesis was supported.

All patients had labral tears, associated with the underlying bone deformities (2 BDDH, six mixed type FAI and one pincer type FAI). A pilot study by Ishihara demonstrated that SIFFH is associated with DDH [8]. A recent case report has shown that SIFFH occurred in patients with overcoverage of acetabular rim that can be implicated with pincer type of impingement [10]. A recent study has shown that eight of nine patients with rapidly destructive hip osteoarthritis associated with SIFFH had inverted labral tears [5]. In our study, our arthroscopic and radiographic findings led us to consider that labral tears associated with underlying bone abnormality can lead to micro-instability, a result of concurrent pathologies including cartilage damage and SIFFH of the femoral head in this case series. It is conceivable that labral tears may contribute to the pathomechanism of SIFFH; however, 100% association in this small series does not establish causation.

For a precollapse SIFFH lesion, non-surgical treatment is usually initially recommended. Several studies have shown that some of SIFFH patients can be managed by non-surgical treatment with survival rate from about 40 to 50% with a high (50–60%) THA conversion rate [6, 9, 14].

Previous studies evaluated the MRI findings of just SIFFH lesion without commenting on concomitant intra-articular pathologies including labral tear, cartilage damage and ligamentum teres injury. In this study, an acetabular labral tear and bony deformities such as FAI and BDDH were found in all patients.

Three of nine patients without fragment fixation could be treated non-operatively for the SIFFH but had extensive labral tears; therefore, arthroscopic labral repair with suture anchors was performed with clinical benefit. Our preferred arthroscopic management for SIFFH lesion with an associated labral tear is a labral repair with FAI correction and capsular closure.

Arthroscopic fixation of SIFFH with HA/PLLA threaded pins is indicated for early phase or precollapse SIFFH. It remains unknown whether this surgery can benefit patients with post-collapse SIFFH or patients with more advanced osteoarthrosis (Tonnis 2 or 3). In this study, the cartilage lesions were classified during diagnostic arthroscopy (Grade 1A in two cases, grade IB in one case, Grade 1C in five cases and Grade 2A in one case). A previous study demonstrated that arthroscopic fragment fixation with HA/PLLA threaded pins can provide excellent clinical outcomes and osteochondral healing for treating elbow osteochondritis dissecans [19]. In fact, HA/PLLA composite materials are widely used for osteochondral injuries with bone defects and used as a strong filler material having osteoconductive properties [18]. A recent case report describes that core decompression with void filter provided excellent clinical outcome for SIFFH [16]. An advantage of this procedure is that post-operative pin removal is not required. Arthroscopic fixation of SIFFH with HA/PLLA threaded pins may facilitate fracture healing via fragment stabilization and/or stimulation of autogenous biologic healing.

In this study, all patients had undergone and failed an initial trial of non-surgical treatment including rest, non-weight-bearing protocol and physiotherapy. Failure was defined as the persistence of symptoms and bone marrow oedema pattern on T2-weighted MRI. Several histological studies have shown that a bone marrow oedema pattern on T2-weighted MRI is frequently found in either SIFFH or osteonecrosis of the femoral head, indicative of fluid exudation due to proliferating fibroblasts and chronic inflammatory cells [11, 23]. Interestingly, the preoperative bone marrow oedema pattern dramatically diminished at one month post-operatively in all cases.

It is suggested that SIFFH in the context of hip instability may be due to the presence of an acetabular labral tear, which is associated with bone deformities. Both open and arthroscopic fragment stabilization allows bone healing and union and improves symptoms of femoral head OCD [13] [17]. Consistent with those studies, osseous union and significant clinical improvement was obtained in all of our patients that underwent arthroscopy with pin stabilization of unstable lesions in precollapse SIFFH.

From the aforementioned evidence, arthroscopic internal fixation of SIFFH with HA/PLLA thread pins along with arthroscopic treatment of associated chondrolabral and bony deformities can enhance the healing process of SIFFH. Thus, the internal fixation of the SIFFH lesion is recommended as part of the comprehensive arthroscopic treatment of recalcitrant precollapse SIFFH afflicted with this condition if the SIFFH lesion is unstable. Fragment fixation is indicated for symptomatic SIFFH lesion grades IB, IC and IIA.

In this study, preoperative physical examinations of all patients showed a positive anterior impingement sign and a positive dial test. MRI as well as arthroscopic findings also showed not only SIFFH, but also acetabular labral tears and bone abnormalities in all patients. Hence, SIFFH was associated bony deformities and acetabular labral tears (see in ``Conclusion’’). Moreover, radiographic union and clinical benefit was observed in all six patients with unstable lesions undergoing arthroscopic surgery including pin stabilization. Our findings indicate that fragment fixation combined with FAI correction, labral preservation and capsular closure can be effective for precollapse SIFFH with unstable lesions, whereas FAI correction, labral preservation and capsular closure procedure without pin fixation can be effective for precollapse SIFFH with stable lesions.

Despite its uncommon prevalence, this study provides clinically relevance by educating health providers about SIFFH as an uncommon, but distinct (via specific MRI criteria) hip condition that may cause recalcitrant symptoms potentially amenable to arthroscopic surgical intervention. The presence of SIFFH should alert the surgeon to the high likelihood of associated bony and labral pathology which may be treated with hip arthroscopy. Moreover, arthroscopic stabilization of unstable precollapse lesions with HA/PLLA threaded pins may yield clinical benefit and bony union.

This study has several limitations. First, this was a retrospective case series study without a control group. Second, a limited number of patients were included in this study. However, SIFFH is a very uncommon condition. Third, short-term follow-up limits conclusions as to the durability of the observed successful outcomes.

## Conclusion

SIFFH is associated with bony deformities and labral tears. Promising outcomes are reported for arthroscopic treatment of precollapse SIFFH with bioabsorbable thread pin stabilization of unstable lesions and treatment of intra-capsular associated pathology in stable ones.
